# An oral M-cell targeted *Lactococcus lactis* vaccine against *Echinococcus multilocularis* infection

**DOI:** 10.3389/fimmu.2025.1683003

**Published:** 2025-10-28

**Authors:** Yang Xiao, Zhao-Hui Luo, Shun-Juan Wang, Yao Dai, Jia-Yu Chen, Jia-Yong Cui, Run-Le Li, Feng Tang

**Affiliations:** 1 Research Center for High Altitude Medicine, Qinghai University, Xining, Qinghai, China; 2 Xiang Xing College of Hunan University of TCM, Yueyang, Hunan, China; 3 NHC Key Laboratory of Echinococcosis Prevention and Control, Lhasa, Tibet, China; 4 Basic Medicine Department of Qinghai University, Xining, Qinghai, China

**Keywords:** *E.multilocularis*, vaccine, M cell, oral, *Lactococcus lactis*

## Abstract

**Background:**

Alveolar Echinococcosis (AE) is a serious infectious disease caused by *Echinococcus multilocularis* (*E.multilocularis,Em*) in the highlands of northwestern China and vaccination is currently the most effective means of preventing *E. multilocularis* infection. However, current vaccines are not sufficiently effective in preventing and controlling Alveolar Echinococcosis.

**Methods:**

In this study, an oral M-cell targeted Lactococcus lactis (*L. lactis*) vaccine (LL-plSAM-GILE) was constructed by adding SAM gene sequence to the epitope vaccine GILE for *E. multilocularis* constructed in our previous study. Mice were orally immunized with LL-plSAM-GILE and their serum antibody levels (ELISA), lymphocyte proliferation (MTS), IFN-γ levels (ELISpot), IL-4 levels (flow cytometry, FCM), T cells (FCM), growth of hepatic cysts (Ultrasound), and weights were measured to evaluate the protective effect of LL-plSAM-GILE.

**Results:**

The L.lactis expression plasmid pNZ8148-SAM-GILE was successfully constructed and electroporated into L.lactis NZ9000, and the recombinant protein was approximately 45 KD. SAM-GILE was expressed on the surface of recombinant L.lactis. LL-plSAM-GILE is effective in targeting Microfold cells. Mice immunized with LL-plSAM-GILE exhibited significantly elevated levels of specific IgG antibodies. Lymphocyte proliferation was enhanced compared to the control group and the NZ9000 group. LL-plSAM-GILE stimulated the production of CD4^+^ and CD8^+^ T cells. Mice immunized with LL-plSAM-GILE secreted more IFN-γ and IL-4. For both primary and secondary infections, oral immunization with LL-plSAM-GILE led to a significant decrease in the diameter and weight of hepatic cysts.

**Conclusions:**

An oral M-cell targeted *L.lactis* vaccine LL-plSAM-GILE with excellent immunogenic and immunoprotective properties has been successfully constructed. This study may provide important theoretical and experimental bases for the prevention and treatment of *E. multilocularis* infection.

## 1 Background

*Alveolar Echinococcosis* (AE) is a chronic parasitic disease caused by *E.multilocularis* infection and it is highly prevalent in many regions of the world but is likely to be neglected. AE will seriously threaten human health and even survival and the mortality rate is over 90% in infected individuals (1). *E. multilocularis* parasitizes the intestines of canids and the larvae can cause echinococcosis in humans and animals. Intermediate hosts (e.g., voles, lemmings, and gerbils) are infected by food or water containing eggs, and end hosts (e.g., foxes, wolves and jackals) are infected by eating the organs of infected animals (2). There are currently no effective control measures for wild end hosts of *E.multilocularis*,. Therefore, a safe, efficient, and low-cost oral vaccine is needed to interrupt the transmission of pathogens by means of immunoprophylaxis. This is of great significance for the prevention and control of vesicular coccidioidomycosis as it can significantly shorten the control process and reduce the cost.

Our previous research has demonstrated that recombinant parasite antigenic proteins expressed in Escherichia coli, such as leucine aminopeptidase (LAP) and EMY162, effectively protected against *E.multilocularis* infection and infiltration in host livers (3). LAP is a metalloprotease of the M17 family and it plays a crucial role in many physiological processes including growth, nutrition and metabolism that are important for the invasion of pathogenic parasites in the host (4). EMY162 is a secreted protein expressed in all life stages of *E.multilocularis* (5). Glucose transporter 1 (GLUT1) is currently the most widely distributed glucose transporter, mediating glucose transport between tissues and the bloodstream (6). The uptake of host-derived glucose by *E.multilocularis* depends on its own glucose transporter, EmGLUT1. Targeting it as an antigen can disrupt the energy supply of *E.multilocularis*, thereby inhibiting their proliferation (7).We have developed a multi-epitope vaccine GILE by combining the antigenic proteins EMY162, LAP, and GLUT1 (7) from *E.multilocularis* (8). The animal experiments revealed that GILE effectively protected against AE infection in mice by activating both humoral and cellular immune responses (8). However, oral vaccines are more practical and feasible compared to injectable vaccines in sparsely populated areas.

*L.lactis* is a food-grade microorganismthat can survive in the gastrointestinal tract of animals and humans without invading or colonizing the mucosal surface of the host (9). Thus, immune tolerance can be avoided due to prolonged stimulation of exogenous antigens (10), and *L.lactis* can also protect exogenous proteins from being degraded by gastrointestinal digestive enzymes and gastric acids, which is beneficial to the functionality and integrity of the exogenous proteins (11).

A prerequisite for the successful development and efficacy of oral vaccines is that their antigens must be phagocytosed by Microfold cells (M cells), and then transported across the mucosal barrier into the mucosa-associated lymphoid tissue (MALT) (12).M cells are located in lymphoid-associated tissues of the intestinal tract and their basement membranes can be invaginated to form a dome-like depression containing a large number of lymphocytes. They have a special structure that enables them to directly ingest foreign antigenic proteins in the intestinal lumen and rapidly present them to the antigen-presenting cells, thereby inducing specific mucosal immunity (13). Therefore, M-cell targeted vaccines may improve antigen presentation efficiency to stimulate a stronger immune response in the host.

In summary, chemical treatment analysis of the cell walls of lactic acidbacteria and Bacillus subtilis showed that the C-terminus of lactic acidbacteria N-acetylmuramidase (AcmA) can bind to the peptidoglycan in bacterial cell walls (14). Additionally, the feasibility of using AcmA as an anchoring protein has been verified in a lactic acidbacteria surface display system (15). Building on these findings, combined with the heteropeptide Mtp—identified in our research group’s previous studies as having M cell-targeting properties—we designed the core component SAM, which features a scientifically rational structure (12). This component is capable of both anchoring to lactic acid bacteria and achieving targeted delivery to M cells.

**Figure 1:**
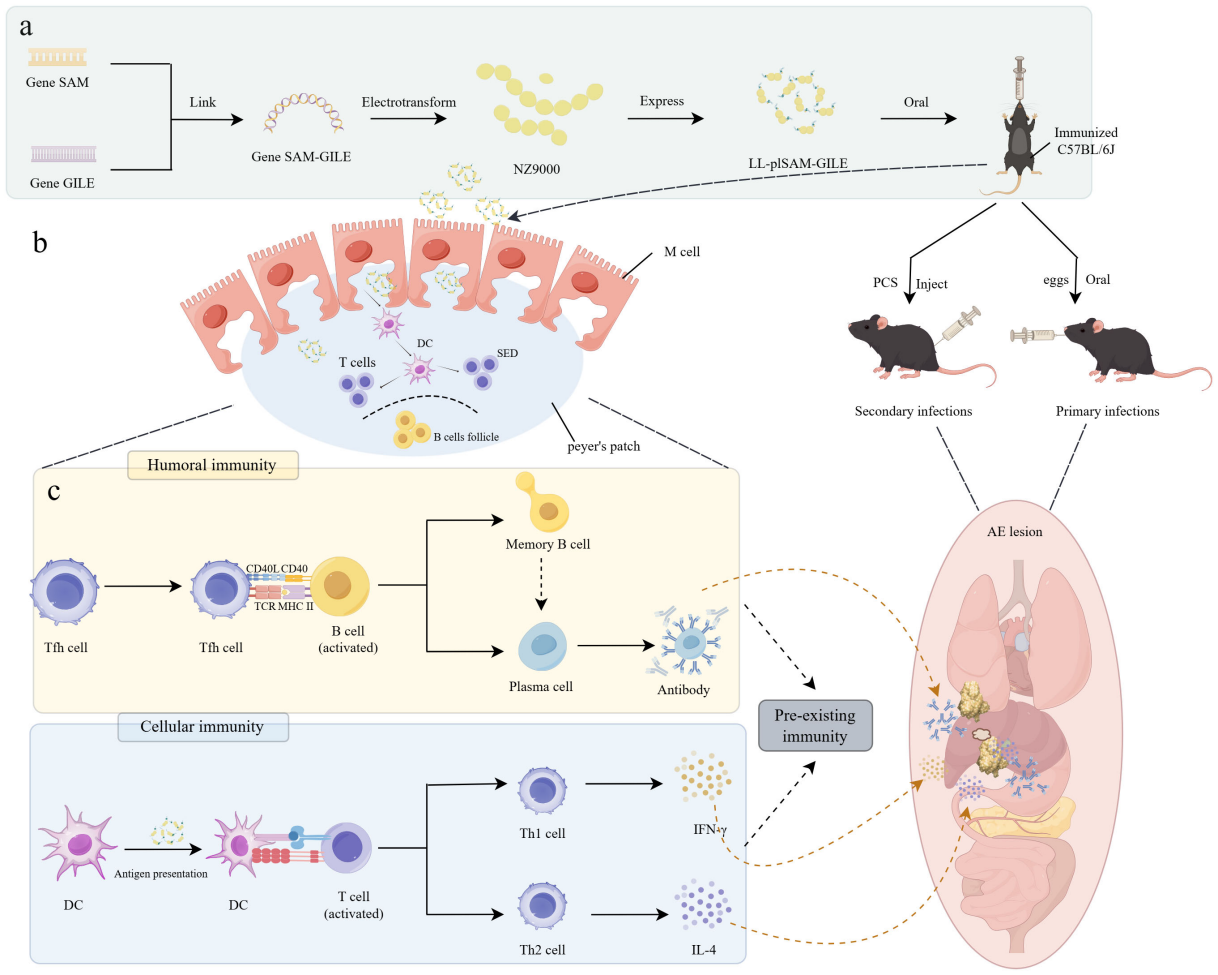
Designing of LL-plSAM-GILE for immunoprophylaxis of *E multilocularis*. **(a)** Engineered strains of *L.lactis* capable of expressing multi epitope peptides and targeting intestinal M cells are developed for oral immunization. **(b)** LL plSAM GILE targets M cells. **(c)** Cyst growth is inhibited through interactions between B and T cells.

In this study, the epitope peptides of *E. multilocularis* with high immunogenicity and the core component SAM were ligated and then introduced onto *L. lactis* NZ9000 to construct recombinant *L.lactis* vaccine LL-plSAM-GILE for oral immunization (**Figure 1A**). This vaccine possesses M cell-targeting capabilities that enable it to specifically direct antigens to M cells and enhance their uptake and antigen presentation (**Figure 1B**), and it may also promote the pre-immunity and humoral and cellular immunity that contribute to more effective control of AE progression while maintaining excellent biological safety (**Figure 1C**).

## 2 Materials and methods

### 2.1 Construction of recombinant LL-plSAM-GILE

**Figure 2:**
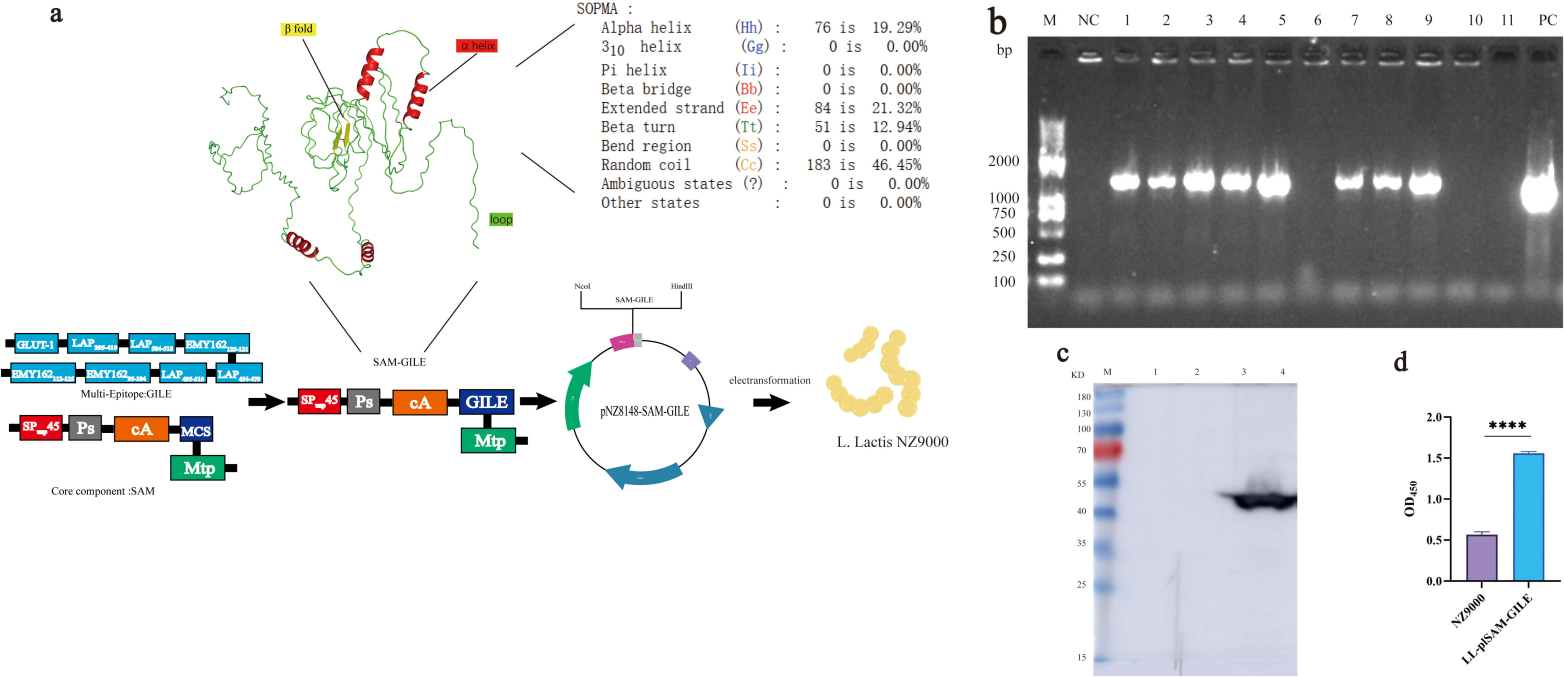
Construction, expression and identification of recombinant *L.lactis* LL-plSAM-GILE. **(a)** Design and construction of recombinant L.lactis LL-plSAM-GILE.SAM: Signal peptide, red portion; leader peptide, gray portion; anchor protein, orange portion; Multiple Cloning Site (MCS), blue portion; M-cell heteropeptide Mtp, green portion. **(b)** PCR. DNA marker (M); Negative Control (NC); Eleven electroporated colonies were picked from the electroporation plates (lane 1-11); Positive Control (PC) **(c)** Western blot. After induction with nisin, the lysate precipitates of LL-plSAM-GILE (lane 4); After induction with nisin, the lysate supernatants of LL-plSAM-GILE (lane 3); The lysate precipitates of LL-plSAM-GILE without induction with nisin (lane 2); The lysate supernatants of LL-plSAM-GILE without induction with nisin (lane 1). **(d)** Whole cell ELISA. The plates were coated with LL-plSAM-GILE (5×10^8^ CFUs/well) and NZ9000 (5×10^8^ CFUs/well). The protein SAM-gile was detected with mouse anti-GILE antiserum and HRP-labeled Goat Anti-Mouse IgG. Data were expressed as mean ± SD, n=6. T-test was performed **(d)**. (*: v.s. NZ9000 group,*P* < 0.05).

SAM-GILE was synthesized by incorporating the SAM gene sequence into the multi-epitope vaccine GILE gene sequence. The secondary and tertiary structure was predicted using SOPMA and Alpha fold, respectively. (Supplementary materials 1) Then, the plasmid pNZ8148-SAM-GILE was obtained by inserting the SAM-GILE gene sequence into the pNZ8148 plasmid. Finally, the recombinant *L. lactis* LL-plSAM-GILE was obtained by transforming the plasmid pNZ8148-SAM-GILE into *L. lactis* NZ9000 (**Figure 2A**), the recombinant protein SAM-GILE will be expressed in the L. lactis strain NZ9000.

### 2.2 Expression of recombinant LL-plSAM-GILE

LL-plSAM-GILE was cultivated and induced for expression of SAM-GILE protein in the presence of 1 ng/mL nisin. Cellular lysate samples were prepared by centrifugation and sonication and then identified by Western blot using Rabbit anti-GILE polyclonal antibody and HRP-labeled Goat Anti- Rabbit IgG (Abcam, UK).

### 2.3 Characterization of surface display

The surface display of the SAM-GILE protein was determined by whole-cell ELISA. ELISA plates were coated with LL-plSAM-GILE and NZ9000 and then washed four times with PBST and blocked with nonfat milk containing 0.1% BSA. Subsequently, mouse anti-GILE antiserum (1: 1000) (Research Center for High Altitude Medicine, Qinghai, China.) was added. After washing four times with PBST, ELISA plates were incubated with HRP-labeled Goat Anti-Mouse IgG. Finally, 100 μL/well of tetramethylbenzidine (TMB) was added, and the reaction was stopped by adding 50 μL/well of 2 M H_2_SO_4_. The absorbance of each well was measured at 450 nm.

### 2.4 Analysis of M-Cell targeting property

**Figure 3:**
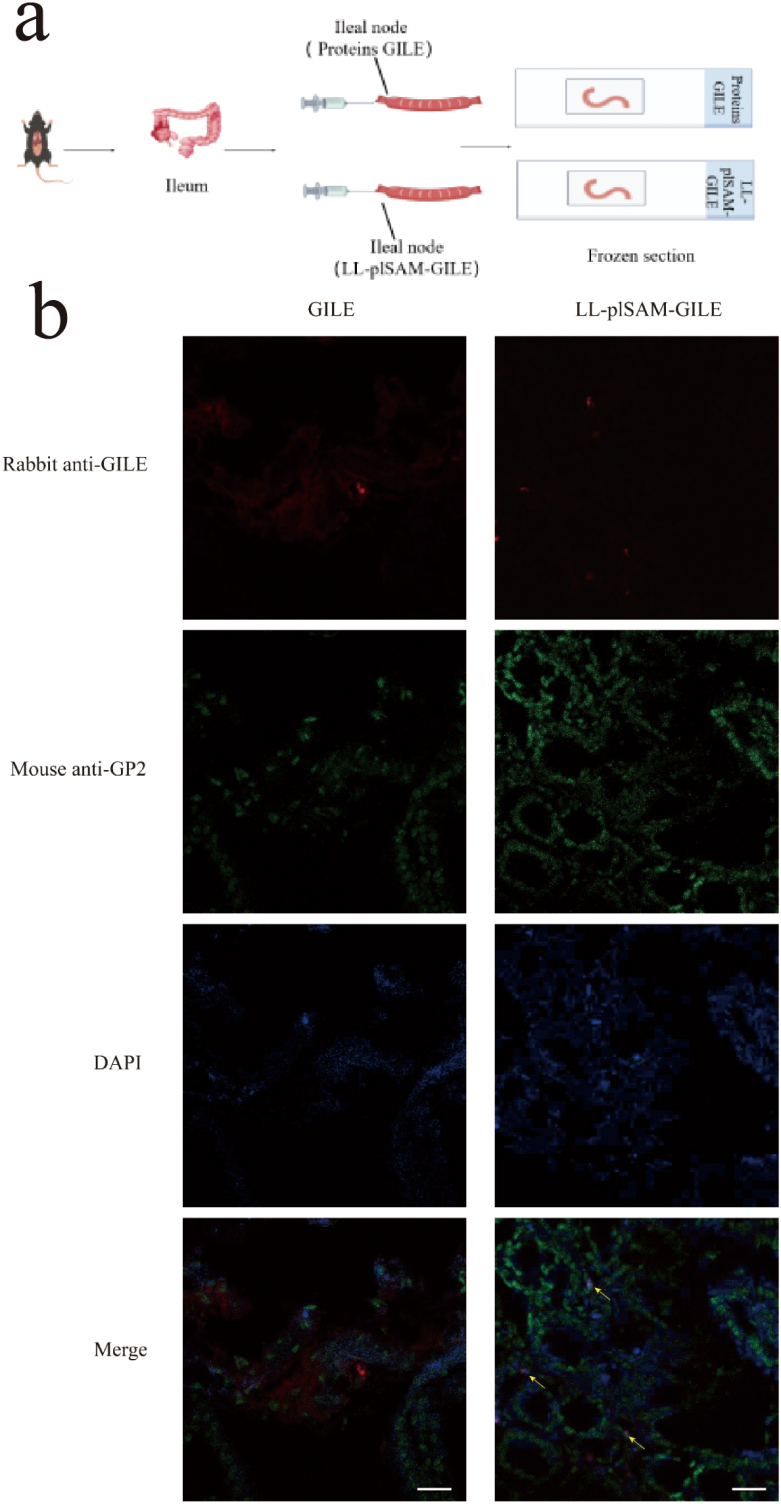
Analysis of M cell-targeting properties. **(a)** For the ileal loop test, LL-plSAM-GILE or GILE proteins were injected into the loops, and frozen sections were prepared. **(b)** For IHC. Yellow arrows indicate co-localization signals for antigens targeting M cells.

The M-cell targeting property of the vaccine was detected by immunofluorescence. Mice (Before immunization) fasted for 16 h were sacrificed and the ileum was intercepted. The ileum was washed with sterile PBS and ligated to form a closed ileocecal tab of approximately 2 cm into the mid-region of the ileum. Then, 100 μL of LL-plSAM-GILE and GILE protein (100μg/ml) were added into the ileal loops respectively. After incubation, the loops were washed, fixed, and then freeze-sectioned (**Figure 3A**). The sections were stained with rabbit anti-GILE antibody and Alexa Fluor 647 Goat anti-rabbit IgG antibody (Abcam, UK). M cells were detected by using Alexa Fluor 488 anti-GP2 monoclonal antibody (MBL, Japan). Nuclei were also stained with DAPI. Finally, anti-fluorescence quencher was added and observed under a confocal microscope.

### 2.5 Immunization program

All animal experiments were performed in compliance with the regulations of the Ministry of Science and Technology of China and approved by the Experimental Committee of Qinghai University (QHDX-2018-09). Six to eight-week-old male C57BL/6J specific pathogen free (SPF) mice were purchased from Jiangsu Huachuang Xinnuo Pharmaceutical Technology Co. (SCXK2019-0010) and fed with sterilized food and water over the 24 h day/night cycle in the Animal Biosafety Level II Laboratory (ABSL-2) of the Research Center for High Altitude Medicine of Qinghai University. Immunization program were performed as described previously with some minor modifications (12).The mice were randomly divided into 3 groups (n=6): LL-plSAM-GILE, NZ9000, and Control. C57BL/6J mice were immunized orally with LL-plSAM-GILE or NZ9000 (3 × 10^9^ CFU) respectively on days 1, 2, 8, 9, 15, 16, 22 and 23, and blood samples were collected at short-tailed intervals every other day to monitor antibody dynamics. One week after the last immunization, mice were sacrificed and their serum and splenocytes were harvested for FCM, MTS, ELISA and ELISPOT testing for immunogenicity.

### 2.6 Detection of antibodies

Serum IgG levels were determined by ELISA. Briefly, ELISA plates were coated overnight at 4°C with 10μg/well of GILE protein. Then, the plates were washed three times with PBST and blocked with 5% skim milk, and serum (1: 1000) was added (100 μL/well) and incubated at 37°C for 1 h. After that, the plates were washed three times with PBST, and horseradish peroxidase (HRP)-conjugated goat anti-mouse antibodies (1: 10000) were added and incubated at 37°C for 1 h. The ELISA plates were washed three times with PBST, and TMB substrate chromogenic solution was added and incubated in dark at room temperature for 5 min, and the reaction was stopped by adding 50 μL/well of 2 M H_2_SO_4_. Finally, the absorbance at 450 nm was detected by a multifunctional microplate reader.

### 2.7 Detection of lymphocyte proliferation

Lymphocyte proliferation was detected by MTS. All mice in immunogenicity experiments were sacrificed one week after the final immunization, and lymphocytes were dissociated from spleen tissues. The spleen tissues were filtered through a cell strainer (Falcon, USA) to obtain single-cell suspensions, and 5 mL of Lympholyte^®^-M Cell Separation Media (CEDARLANE Canada) was added. Lymphocytes were isolated by centrifugation (25°C, 20 min, 1000–1500 g),. After that, the liquid was separated into three layers (plasma, lymphocyte and red blood cell) from top to bottom, and lymphocytes were transferred to another tube and washed three times with serum-free RPMI-1640 medium. The lymphocyte suspension (1×10^6^) mixed with GILE protein (10 μg/mL) was added to 96-well plates (200 μL/well) and incubated at 37°C for 60 h in 5% CO_2_. Next, MTS was added (20 μL/well) and incubated at 37°C for 4 h in 5% CO_2_. Finally, the absorbance at 490 nm was detected by a multifunctional microplate reader.

### 2.8 Detection of cytokines

Lymphocytes were prepared as described in Section 7. The levels of interleukin-4 (IL-4) and interferon-gamma (IFN-γ) were detected using the ELISpot Kit (Biotopped, USA) according to the manufacturer’s instructions. The ELISpot plates were washed four times with sterile 1×PBS (200 μL/well), and RPM1–1640 medium with 10% fetal bovine serum was added (200 μL/well) and incubated for 0.5 h at 37°C in 5% CO_2_. Then, the plates were emptied and the splenocyte suspension (2.5×10^6^) mixed with GILE protein (10 μg/mL) was added (100 μL/well) and incubated for 18–48 h at 37°C in 5% CO_2_. Then, R4-6A2-biotin was diluted to 1 μg/ml in PBS containing 0.5% fetal calf serum (PBS-0.5% FCS), and incubated (100 μl/well) for 2 h at room temperature. Next, Streptavidin-ALP (1: 10000) was diluted in PBS-0.5% FCS and incubated (100 μl/well) for 1 h at room temperature. BCIP/NBT-plus was filtered through a 0.45 μm filter and added at 100 μl/well and allowed to develop until distinct spots appeared. Finally, the spots were counted using an ELISPOT automatic platereader.

FCM was performed to verify the detection of cytokines. The lymphocyte suspension (1×10^6^) mixed with GILE protein (10 μg/mL) was added to 24-well plates (1 mL/well) and incubated for 1 h at 37°C in 5% CO_2_. The same amount of PBS and cell stimulation cocktail (BD, USA) were added as negative control and positive control, respectively. Then, Brefeldin A Solution was added and incubated for 6.5 h at 37°C in 5% CO_2_. After that, Anti-Mouse CD4 PE-Cyanine7 (BD, USA) was added and incubated in dark for 20 min at room temperature. The supernatant was removed by centrifugation (4°C, 1000–1500 g,10 min). The Fix/Perm solution (BD, USA) was added and incubated in dark for 30 min at room temperature. After centrifugation (4°C, 500 g, 10 min), 1×Permeabilization Buffer mixed with Anti-Mouse IL-4 APC (BD, USA) and Anti-Mouse IFN-γ FITC (BD, USA) was added and incubated in dark for 30 min at 4°C. The supernatant was removed by centrifugation (4°C, 500 g, 10 min). Lymphocytes were washed three times with PBS-0.5% FCS and then re-suspended with PBS-0.5% FCS. Finally, the fluorescence was detected by FCM (FACS AsiaIII^®^, ty20204251).

### 2.9 Detection of CD4^+^ and CD8^+^ T cells

FCM was performed to verify the detection of CD4^+^ and CD8^+^ T cells. FCM were performed as described previously (8). Lymphocyte specific antibodies were used to label lymphocytes (1×10^6^). Specifically, CD4^+^ T cells were labeled with Anti-Mouse CD4 FITC (BD, USA) and CD8^+^ T cells were labeled with Anti-Mouse CD8a APC (BD, USA). At last, the levels of CD4^+^ and CD8^+^ T cells were detected by flow cytometry.

### 2.10 Challenge experiment

Challenge experiment were performed as described previously with some minor modifications (8, 16). Primary infection was modeled to evaluate the protective efficacy of the vaccine. Mice were immunized as described above. One week after the last immunization, mice were orally gavaged with 100μL of the suspension containing 1000 *E. multilocularis* eggs (Qinghai Provincial Institute of Endemic Disease Prevention and Control, Qinghai, China.). After four months, mice were sacrificed to evaluate the protective effect of the vaccine.

Secondary infection was modeled to evaluate the protective efficacy of the vaccine. Again, mice were immunized as described above. One week after the last immunization, 200 μL of the mixture containing 2,000 *E.multilocularis* protoscoleces (Basic Immunity Laboratory for Zoonoses, Qinghai University, China.) was injected into the peritoneal cavity of the immunized mice. After four months, mice were sacrificed to evaluate the protective effect of the vaccine.

### 2.11 Evaluation of the protective effect of LL-plSAM-GILE

Mice were anesthetized with 1.5% isoflurane in O_2_ on a specially designed heated bed for measurement of the cyst area in the epigastrium. Ultrasonography was performed using a FUJIFILM Vevo^®^ Ultrasonic imager for small animals; and MRI was performed using a BioSpec^®^ MRI scanner (Bruker PharmaScan 70/16 USR) and 7.0T MR Scanning system for small animals. Then, mice were sacrificed and their cysts were isolated and weighed.

### 2.12 Statistical analyses

All statistical analyses were performed using SPSS28.0 software. Data were expressed as mean ± SD. Comparisons between two groups were performed using an independent sample t test. *P* < 0.05 indicated statistically significant differences (* *P* < 0.05; ** *P* < 0.01; *** *P* < 0.001; ns no statistical significance).

## 3 Results

### 3.1 Construction of recombinant *L. lactis* LL-plSAM-GILE

The secondary structure of SAM-GILE was predicted online using SOPMA, which consisted of α helix (19.29%), β fold (21.32%), β turn (12.94%), and random coil (46.45%) (**Figure 2A**). SAM-GILE might have good immunogenicity because of the presence of more β turn and random coil (**Figure 2A**). A bright band was clearly observed at 1300 bp (**Figure 2B**) on lane 1, 2, 3, 4, 5, 7, 8 and 9, respectively, and there is a good agreement between observed and expected sequences size. The PCR results suggested that recombinant *L.lactis* vaccine LL-plSAM-GILE was successfully prepared.

### 3.2 Expression of recombinant *L. lactis* LL-plSAM-GILE

The Western blot results confirmed that the fusion protein SAM-GILE could be detected by mouse anti-GILE polyclonal antibody. LL-plSAM-GILE could produce the fusion protein SAM-GILE (45 kDa) (**Figure 2C**).

### 3.3 Surface display

The specific reactivity to mouse anti-GILE antiserum was detected in the LL-plSAM-GILE group (1.558 ± 0.0212) but not in the NZ9000 group (0.565 ± 0.0364). The finding indicated that the surface of LL-plSAM-GILE was capable of displaying the recombinant antigenic protein SAM-GILE (**Figure 2D**).

### 3.4 M-cell targeting properties

Immunohistochemistry (IHC) was performed to identify whether LL-plSAM-GILE had M cell-targeting properties. LL-plSAM-GILE and GILE protein was injected into the ileal loops respectively. The results revealed that the groups treated with LL-plSAM-GILE showed a higher overlap with M cells in Peyer’s patches compared to the control group treated with GILE protein (**Figure 3B**), suggesting that LL-plSAM-GILE had better M-cell targeting properties due to the presence of the SAM component.

### 3.5 Immunogenicity of LL-plSAM-GILE

#### 3.5.1 Serum-specific antibody levels of LL-plSAM-GILE

**Figure 4:**
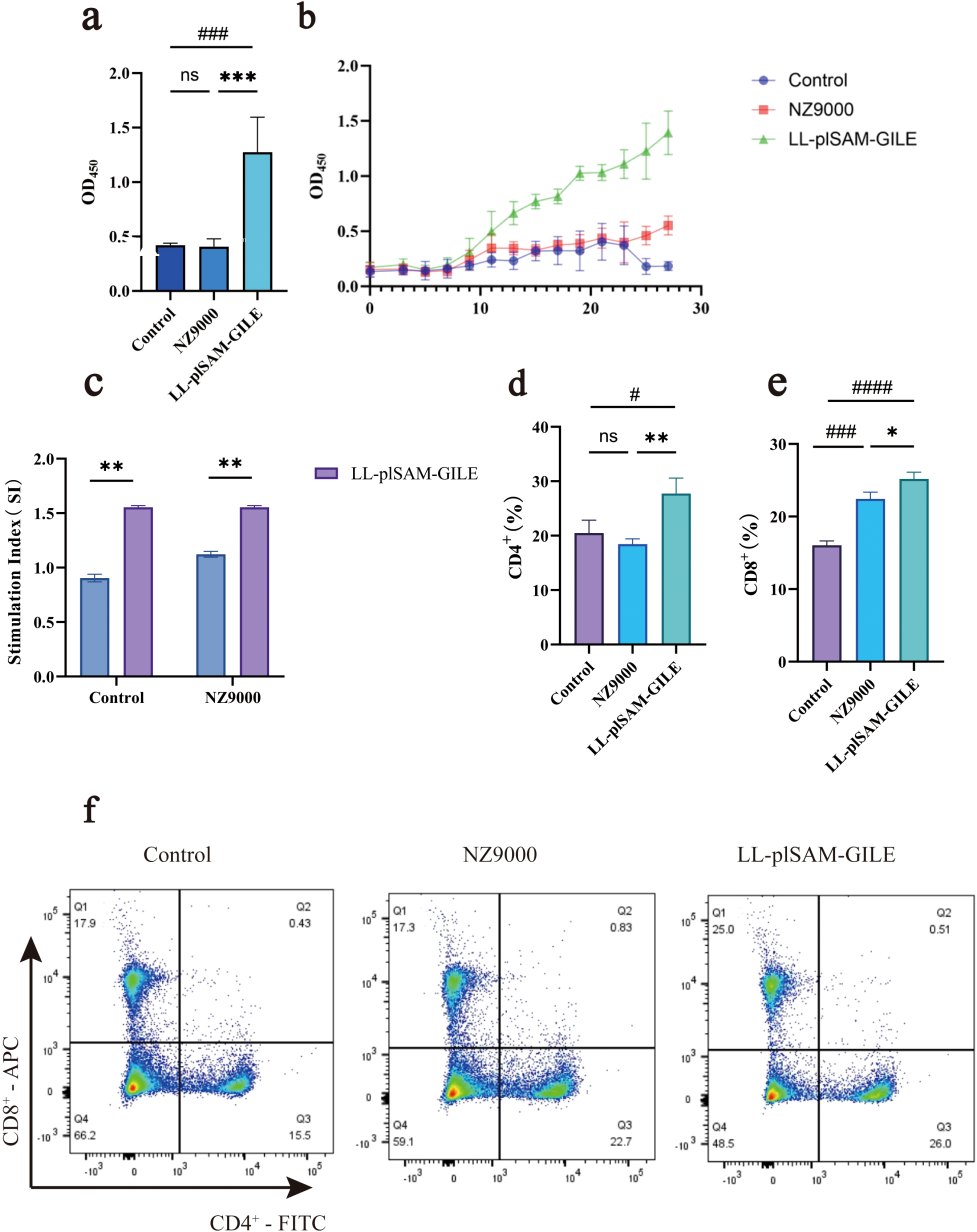
Recombinant L.lactis LL-plSAM-GILE induces humoral immune response and lymphocyte proliferation. **(a)** The total IgG levels at week four after immunization by ELISA. **(b)** Dynamic monitoring curve of serum IgG antibody in mice. **(c)** Mouse spleen lymphocyte proliferation by MTS. **(d–f)** CD4^+^ and CD8^+^ T cells were detected by flow cytometry in mouse spleen. Data were expressed as mean ± SD, n=6. One-way ANOVA was performed **(a–e)**. (#: v.s. Control group, *P* < 0.05; *: v.s. NZ9000 group, *P* < 0.05).

Mice were sacrificed one week after the final immunization and their serum was isolated for detection of IgG by indirect ELISA. Compared to the Control group (0.420 ± 0.018) and NZ9000 group (0.407 ± 0.071), LL-plSAM-GILE group (1.275 ± 0.321) exhibited a stronger humoral immune response (**Figure 4A**) and a significantly higher level of serum specific IgG. The dynamic changes of IgG were detected by indirect ELISA (**Figure 4B**). Compared to the Control group and NZ9000 group, the IgG level was increased in mice immunized with LL-plSAM-GILE. However, the IgG levels of the Control group and NZ9000 group remained low throughout the experiment.

#### 3.5.2 Detection of spleen lymphocyte proliferation by MTS

To test the capacity of LL-plSAM-GILE to induce specific lymphocyte responses, splenic lymphocytes were cultured with GILE protein. LL-plSAM-GILE group (1.554 ± 0.015) displayed significantly higher proliferation than NZ9000 group (1.112 ± 0.034) or Control group (0.905 ± 0.035) (**Figure 4C**).

#### 3.5.3 Detection of CD4^+^ and CD8^+^ T-Cell by FCM

The number of CD4^+^ and CD8^+^ T cells of the LL-plSAM-GILE group (CD4^+^: 27.73 ± 2.857; CD8^+^: 25.20 ± 0.916) was significantly higher than that of the Control group (CD4^+^: 20.47 ± 2.380; CD8^+^: 16.03 ± 0.611) and NZ9000 group (CD4^+^: 18.43 ± 0.986; CD8^+^: 22.43 ± 0.929) (**Figures 4D-F**). Notably, the number of CD8^+^ T cells was also increased in NZ9000-immunized mice compared to the control group, suggesting that NZ9000 could be used as an adjuvant to enhance LL-plSAM-GILE induced immune responses.

#### 3.5.4 Detection of cytokines by ELISpot and FCM

**Figure 5:**
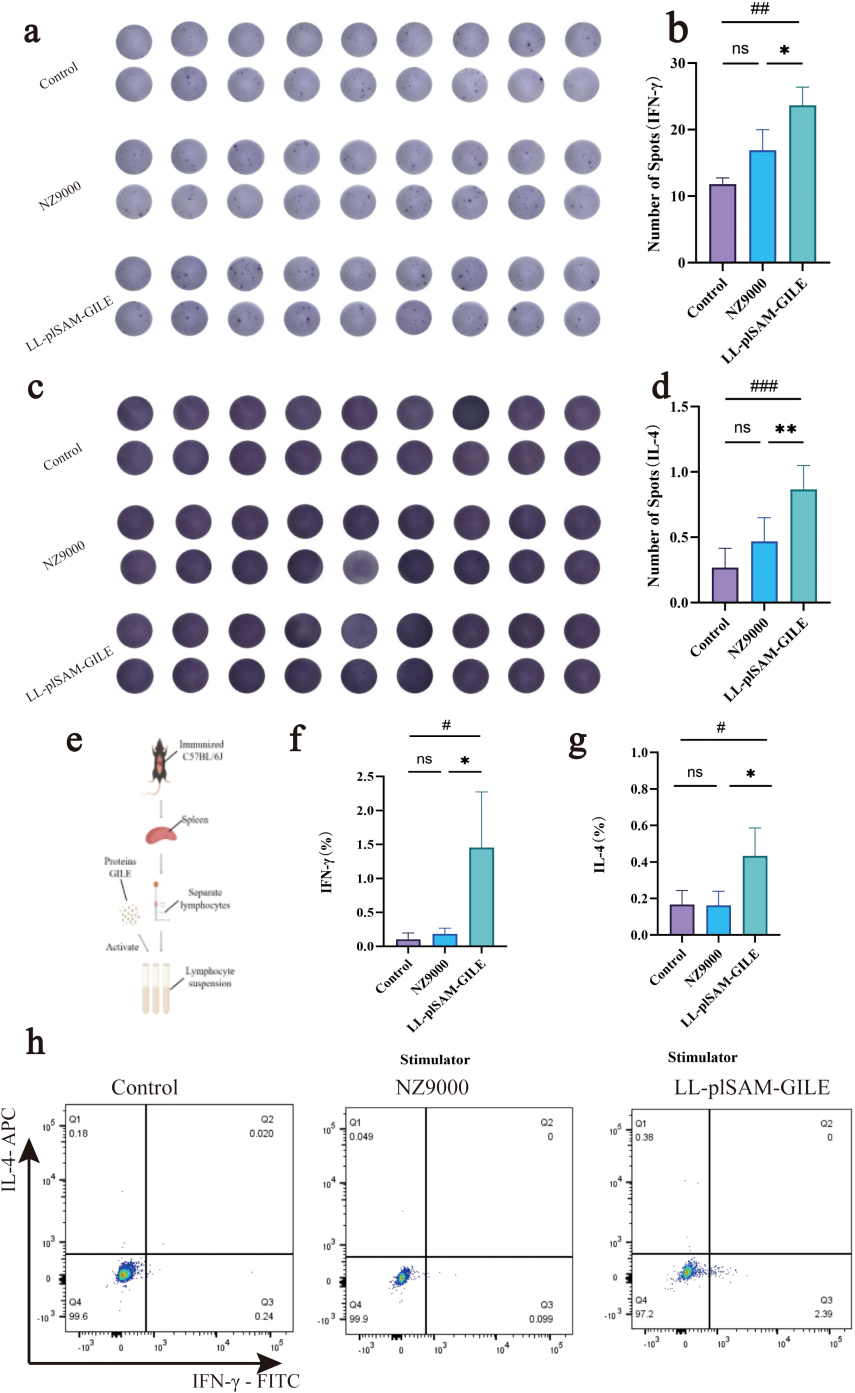
Recombinant L.lactis LL-plSAM-GILE induces cell immune response. **(a–d)** ELISpot analysis of IFN-γ and IL-4 of splenocytes. **(e)** Stimulation of splenocytes to secrete cytokines. **(f–h)** Detection of cytokine IFN-γ and IL-4 secretion by flow cytometry. Data were expressed as mean ± SD, n=6. One-way ANOVA was performed **(b, d, f, g)**. (#: v.s. Control group, *P* < 0.05; *: v.s. NZ9000 group, *P* < 0.05).

GILE proteins were used to stimulate spleen lymphocytes to secrete cytokines. The Th1-type cytokine IFN-γ and the Th2-type cytokine IL-4 were detected by ELISpot (**Figures 5A-D**) and FCM (**Figures 5E-H**), respectively. The levels of IL-4 and IFN-γ were significantly higher in the LL-plSAM-GILE group than in the Control group or the NZ9000 group.

### 3.6 Protective effect of LL-plSAM-GILE

**Figure 6:**
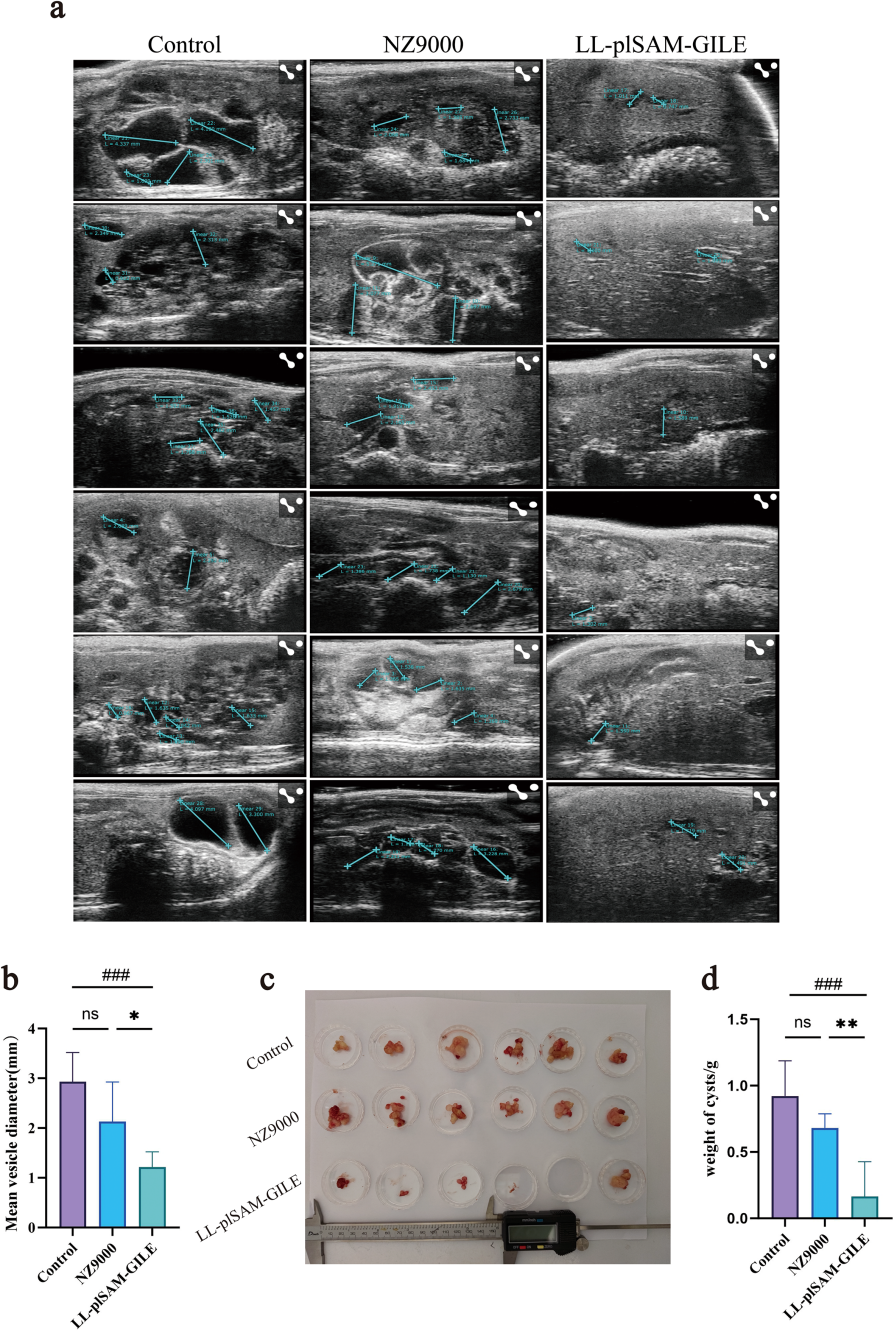
Recombinant L.lactis LL-plSAM-GILE inhibited cyst growth in secondary infection. **(a, b)** Representative Ultrasound images of mouse liver four month after E.multilocularis challenge. **(c, d)** The weight of the hydatid body four month after *E.multilocularis* challenge. Data were expressed as mean ± SD, n=6. One-way ANOVA was performed **(b, d)**. (#: v.s. Control group, P<0.05; *: v.s. NZ9000 group, *P* < 0.05).

**Figure 7:**
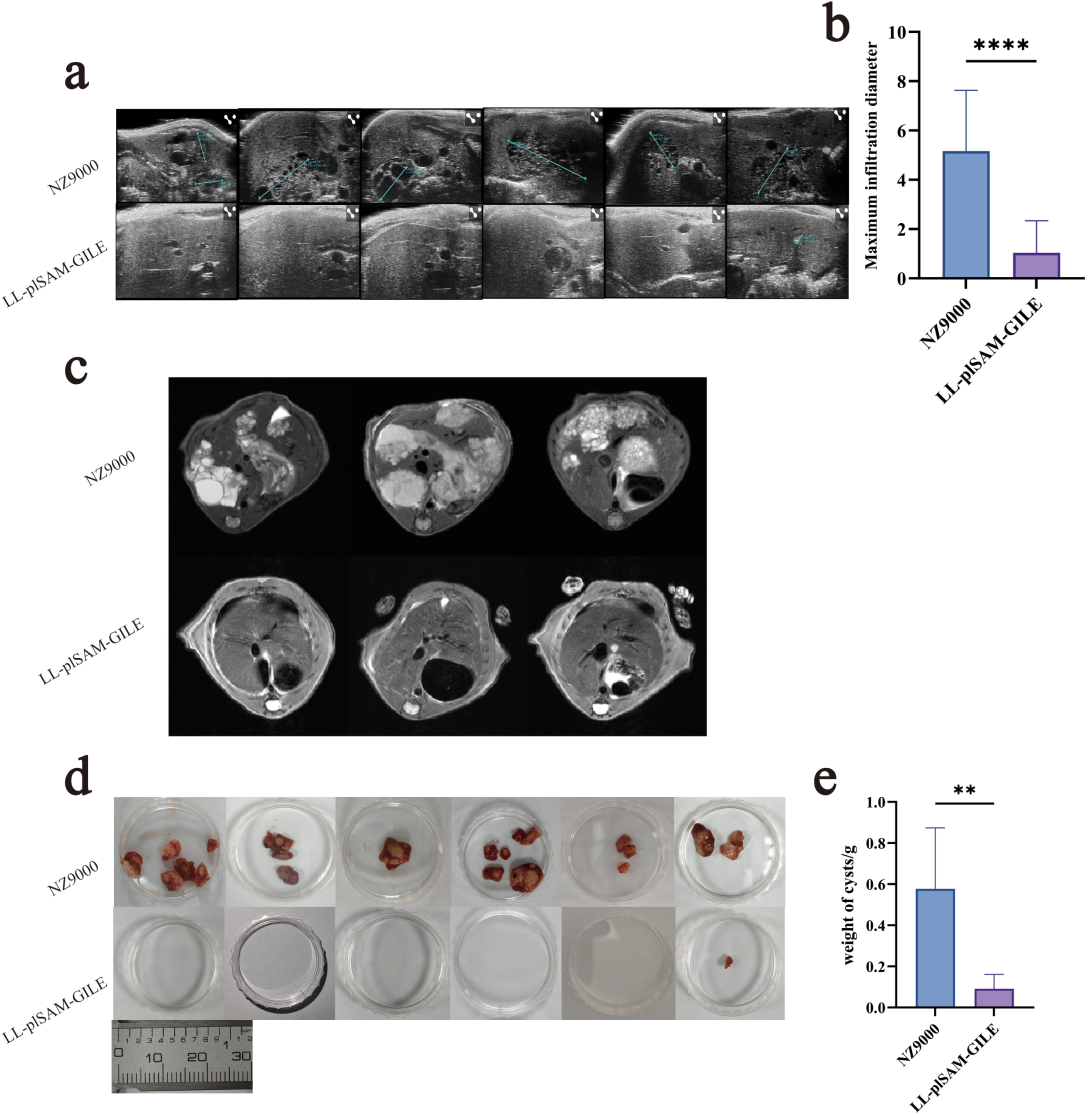
Recombinant L.lactis LL-plSAM-GILE inhibited cyst growth in primary infection. **(a, b)** Representative Ultrasound images of mouse liver four month after E.multilocularis challenge. **(c)** Representative MRI images of mouse liver four month after E.multilocularis challenge. **(d, e)** The weight of the hydatid body four month after E.multilocularis challenge. Data were expressed as mean ± SD, n=6. T-test was performed. **(b, e)**. (v.s. NZ9000 group, *: *P* < 0.05, **: *P* < 0.01, ***: *P* < 0.001 and ****: *P* < 0.0001).

The growth of hepatic cysts of the secondary infection model was evaluated by Ultrasound. The cysts of the LL-plSAM-GILE group were much smaller than that of the Control group and NZ9000 group (**Figures 6C, D**). The cysts of the LL-plSAM-GILE group (0.166 ± 0.261) were lighter compared to the Control group (0.922 ± 0.265) and the NZ9000 (0.682 ± 0.106) group (**Figures 6A,B**). The growth of hepatic cysts of the primary infection model was evaluated by Ultrasound and MRI. The ultrasonic results showed that (**Figures 7A, B**) many cysts invaded into liver tissues in NZ9000-immunized mice and their sizes were significantly increased compared to the LL-plSAM-GILE group (NZ9000: 5.16 ± 2.47; LL-plSAM-GILE: 1.03 ± 1.30). MRI (including T1, T2 and DWI) was performed to indicate the anatomical position, *E. multilocularis* lesions, and the invasion of cysts and metacestodes in the liver, respectively. The T2 sequence results (**Figure 7C**) showed that cysts grew rapidly and almost occupied the whole liver in the NZ9000 group; while the LL-plSAM-GILE group showed little or no lesions in the liver. Then, cysts were isolated from thoracic, abdominal, and subcutaneous tissues and weighed. The cysts of the LL-plSAM-GILE group (0.091 ± 0.070) were lighter compared to the NZ9000 group (0.577 ± 0.296) (**Figures 7D, E**). These results suggested that LL-plSAM-GILE could protect against *E. multilocularis* infection.

## 4 Discussion

AE is one of the most prevalent parasitic diseases globally. Clinically, it exhibits tumor-like progression, characterized by invasive growth into the adjacent tissues of the host (3). Vaccination represents an effective broad-spectrum intervention strategy against AE: it not only elicits a specific immune response in the host but also exerts inhibitory effects on the growth and metabolic activities of AE cysts. Natural infections of *E. granulosus* primarily occur in large mammals, such as domesticated cattle and sheep, which can be managed through the planned use of anthelmintic drugs, along with the promotion of slaughter hygiene and health education (17). In contrast, *E.multilocularis* predominantly infects wild animals, serving as both intermediate and final hosts, making it more challenging to control than *E.granulosus*. *E.multilocularis* imposes a considerable burden on human and veterinary health and is responsible for high economic losses, in particular in the case of AE. Because *E.multilocularis* primarily infects hosts through ingestion, *L. lactis* as an oral vaccine for *E.multilocularis* infection may be a more effective and practical vaccine for the prevention and control of AE.

The activation of the immune system is a synergistic process characterized by “recognition of conformational epitopes by B cells and recognition of linear epitopes by T cells”. Both β turn and random coil exhibit distinct advantages in these two stages, which can simultaneously enhance the activation of B cells and the antigen-presenting efficiency of T cells, thereby mediating the “dual-signal synergistic amplification” effect. Preliminary experimental data have shown that recombinant proteins with increased content of β turn and random coil can induce enhanced immune responses (8). Therefore, in the study, extra β turn and random coil were rationally incorporated into the recombinant protein, and the strong immunogenicity of the recombinant protein was further validated in subsequent animal-based experimental models.

*L. lactis* are acknowledged as food-grade microorganisms and play a crucial role as probiotics in the mammalian gut (18–20). As probiotics readily live in the digestive tract without harming it and retain tight contact with the epithelium (21–23), *L. lactis* is a good candidate for the delivery of heterologous proteins in foods because of its many advantages such as safety, simplicity, affordability, easy preparation, and practicality (24). Recently, a number of *L.lactis* -based vaccines have been reported (25–27), including recombinant LL-TSOL18 vaccine expressing the Taenia solium TSOL18 protein, recombinant LL-PpSP15 vaccine expressing the Leishmania major PpSP15 protein, and recombinant LL-OMP31 vaccine expressing the Brucella melitensis OMP31 protein. These vaccines have been demonstrated to have mixed Th1 and Th2 responses following multiple oral or subcutaneous injections in BALB/c mice. L. lactis is recognized as a common oral probiotic, and L. lactis-vectored vaccines may become an optimal choice for oral vaccination. However, there is no report of a recombinant *L. lactis* vaccine of *E.multilocularis* in the parasite field.

However, oral immunization of of *L.lactis* remains a challenging problem, such as induction of immune tolerance and suboptimal antigen presentation. M cells are a specialized type of epithelial cells in the intestine, which are characterized by a lack of microvilli and a thick mucus layer. This unique morphology results in the invagination of the basement membrane, forming a structure reminiscent of a “small capsule”. It also greatly enhances the ability of M cells to uptake foreign substances and transport them efficiently over distance and time, thereby facilitating the internalization of particulate antigens, bacteria, and viruses, as well as the rapid activation of host mucosal immune responses (12, 28, 29). Additionally, Guo Le et al. (12) validated that the recombinant *L. lactis*-based vaccine against Helicobacter pylori designed by them possesses excellent M cell-targeting capability, which verifies the reliability of their established methodology. In light of this, IHC was also employed in the study for the identification of M cell targeting. In this study, M-cell targeted peptide SAM was fused to the GILE gene to generate the fusion proteins known as SAM-GILE. These fusion proteins were designed to be displayed on the surface of *L. lactis* and to bind specifically to rabbit antisera directed against the GILE protein. Additionally, the distribution of LL-plSAM-GILE overlapped with M-cells in mouse ileal tissue sections. The results indicated that the recombinant *L.lactis* LL-plSAM-GILE was successfully prepared and demonstrated good M-cell targeting properties. The fusion of the core component SAM with the GILE gene proved effective in enhancing the stability and antigenicity of the protein in the intestine. Thus, it is feasible to construct an oral *L. lactis* vector for the subunit vaccine GILE. These results provide important experimental evidence for further studies on the immune response induced by the LL-plSAM-GILE vaccine in immunized mice.

Lymphocyte proliferation is an important indicator of cellular immunity. The splenic lymphocytes collected from orally immunized mice exhibited a strong proliferative response following antigen stimulation, suggesting that recombinant LL-plSAM-GILE may effectively induce specific cellular responses. This finding is consistent with our subsequent observation of elevated levels of specific CD4^+^ and CD8^+^ T cells in mice immunized with orally administered LL-plSAM-GILE.

Activated CD4^+^ T cells proliferate and differentiate into effector Th cells. It is known that IFN-*γ*, IL-2, and TNF-*α* are indicators of Th1 response, which promote the production of IgG2a and IgG2b; whereas IL-4, IL-5, and IL-10 are indicators of Th2 response, which promote the production of IgG1 and IgG3 (25). An imbalance between Th1-type and Th2-type immune responses, along with a transition from Th1 (protective) to Th2 (non-protective) immune response, may play a crucial role in facilitating immune evasion by parasitic organisms (30–32). Our results indicate that stimulation with the GILE protein markedly elevated the production of IFN-γ and IL-4 in the splenic lymphocytes of mice immunized with oral LL-plSAM-GILE. The antibody response exhibited a notable increase in IgG levels in mice immunized with oral LL-plSAM-GILE. Humoral immunity plays a predominant role in combating parasitic infection, with IgG being the most prevalent antibody in serum and the most enduring and critical antibody within the humoral immune response (33). These findings imply that LL-plSAM-GILE induces a mixed Th1-Th2 immune response and a specific humoral response within the host, which may initiate a robust and sustained immune reaction and therefore enhance the host’s ability to control *E.multilocularis* infection.

Challenge tests were performed to evaluate the efficacy of oral vaccination. In the present study, two animal models were established by intraperitoneal injection of protocephalic nodes and oral administration of eggs, respectively. Notably, the *in vivo* lesions in mice with secondary infection were found to be globular cysts, mostly in the liver and abdominal cavity rather than in a single organ. These cysts were not tightly connected to the liver tissue and some of them could be easily peeled off. These results are in line with previous findings (8, 34). However, the lesions in mice with primary infection grew infiltratively in the liver and they first appeared in the liver and then grew upward along the diaphragm, similar to the natural route of infection (3, 5, 8). A similar phenomenon was observed in mice with primary infection. Additionally, the ultrasonic results revealed that in mice with secondary infection, the diameters of liver cysts were significantly smaller in the LL-plSAM-GILE group compared to both NZ9000 and control groups. The cyst weight was lowest in the LL-plSAM-GILE immunized group following the dissection of the mice. More importantly, primary infected mice in the LL-plSAM-GILE group also demonstrated minimal cyst diameter and weight.

But there still have some deficiencies in this study. This study primarily evaluated the systemic immunogenicity of the vaccine. The capacity of the vaccine to induce intestinal mucosal immunity requires further confirmation in future studies by directly measuring mucosal biomarkers such as fecal IgA. However, the observed potent systemic immunity, particularly the Th1and Th2 response, provides optimistic clues for its potential protective role at mucosal sites.

## 5 Conclusions

In summary, a *L.lactis* vaccine LL-plSAM-GILE against *E.multilocularis* was constructed, based on a designed M cell-targeting surface display system for *L.lactis*. *L. lactis* LL-plSAM-GILE could display the SAM-GILE antigen on the surface of bacteria, and LL-plSAM-GILE had an enhanced M cell-targeting property. Oral immunization with LL-plSAM- GILE could stimulate antibodies against *E.multilocularis* and *E.multilocularis*-specific CD4^+^ and CD8^+^ T cells, thus providing protective immunity against *E.multilocularis* infection. The mice immunized orally with LL-plSAM-GILE exhibited minimal cyst diameter and weight. In conclusion, the recombinant *L.lactis* LL-plSAM-GILE developed in this study effectively activates the host’s immune system to combat *E.multilocularis* infection. This research provides valuable insights into the development of *E.multilocularis* vaccines.

## Data Availability

The original contributions presented in the study are included in the article/**Supplementary Materials**. Further inquiries can be directed to the corresponding authors.
