# Correction: An oral M-cell targeted *Lactococcus lactis* vaccine against *Echinococcus multilocularis* infection

**DOI:** 10.3389/fimmu.2025.1741460

**Published:** 2025-11-19

**Authors:** Yang Xiao, Zhao-Hui Luo, Shun-Juan Wang, Yao Dai, Jia-Yu Chen, Jia-Yong Cui, Run-Le Li, Feng Tang

**Affiliations:** 1 Research Center for High Altitude Medicine, Qinghai University, Xining, Qinghai, China; 2 Xiang Xing College of Hunan University of TCM, Yueyang, Hunan, China; 3 NHC Key Laboratory of Echinococcosis Prevention and Control, Lhasa, Tibet, China; 4 Basic Medicine Department of Qinghai University, Xining, Qinghai, China

**Keywords:** *E.multilocularis*, vaccine, M cell, oral, *Lactococcus lactis*

Authors “Yang Xiao and Zhao-Hui Luo” were erroneously omitted as equal contributing authors. The original version of this article has been updated.

Affiliation 3 “NHC Key Laboratory of Echinococcosis Prevention and Control, Lhasa, Tibet, China” was erroneously given as “Tibet Key Laboratory of Echinococcosis, Lhasa, China”. The original version of this article has been updated.

An incorrect number was provided for “Open Research Projects of the National Health Commission Key Laboratory of Echinococcosis Prevention and Control”. The correct number is “No.2023WZK1003”.

A second grant number was erroneously omitted for funder “Natural Science Foundation of China”. The additional number is “Grant No. 81860299”, directly after the existing “Grant No.32360192”.

The original version of this article has been updated.

