# Supplementary material for: An oral M-cell targeted Lactococcus lactis vaccine against Echinococcus multilocularis infection
Source: Front Immunol. 2025 Oct 28;16:1683003. doi: 10.3389/fimmu.2025.1683003 (PMC12602529; doi:10.3389/fimmu.2025.1683003)
Supplement: Supplementary file 1 [file DataSheet1.pdf]

An oral M-cell targeted *Lactococcus lactis* vaccine against *Echinococcus multilocularis* infection

Yang X & Feng Tang.et al.

Supplemental Stability Data

1. Amino acid sequence of the recombinant protein SAM-GILE

```
        10        20        30        40        50        60        70
         |         |         |         |         |         |         |
IMDNGPAHKQGVASDKKIMDNGPAHKQGVASDKKGAPLLMFTSMYILPRKKGAPLLMFTSMYILPRKKQE
eecccccchtccchhheeecccccccteeecccccccceeeetceeeeccttcceeecttcceeecccccc
GEQNDKSTEAAEEAYQKATSKKQEGEQNDKSTEAAEEAYQKATSKKTGFIRTLAPGEDGADRKKTGFIRT
ccccchhhhhhhhhhhhhhccchtcccccchhhhhhhhhhhhhhctttttceeeecttcccccccccccheee
LAPGEDGADRKKAFMIAASGLDEHGLGSGSAFMIAASGLDEHGLGSGSAGAIDVLPTGAPLLMFGSAGAI
ecttccctttccchheeeehtccchtcccccccheeeecccccetcccccttceeecccccceeeeccttce
DVLPTGAPLLMFGSATSLAEAELSVTHPGSATSLAEAELSVTHPGSQEGEQNDKSTEAAEEAYQKATSGS
eeecttcceeeecccchhhheeeecctttccceecttcheecccccccccccchhhhhhhhhhhhcttc
QEGEQNDKSTEAAEEAYQKATSGSKLTLEGLKPSTFYEVGSKLTLEGLKPSTFYEVGSALKGDSEVYKGS
ccthccchhhhhhhhhhhhhhcttceeeeeetccttceeeeeeccccettccttcheehhhhhttcceecttc
ALKGDSEVYKGSLAPGEDGADRAGGFAGSLAPGEDGADRAGGFA
ceecccccceeeeccttcccccccccccccccttcccccccccee
```

2. Protein secondary structure prediction of the recombinant protein SAM-GILE

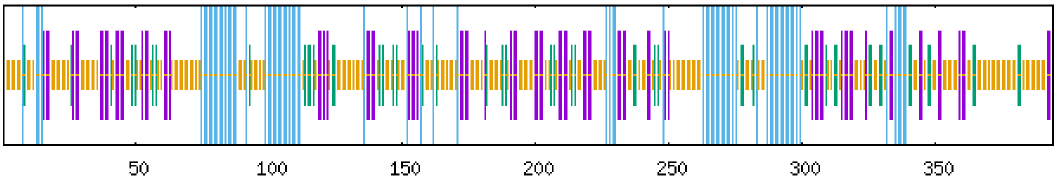

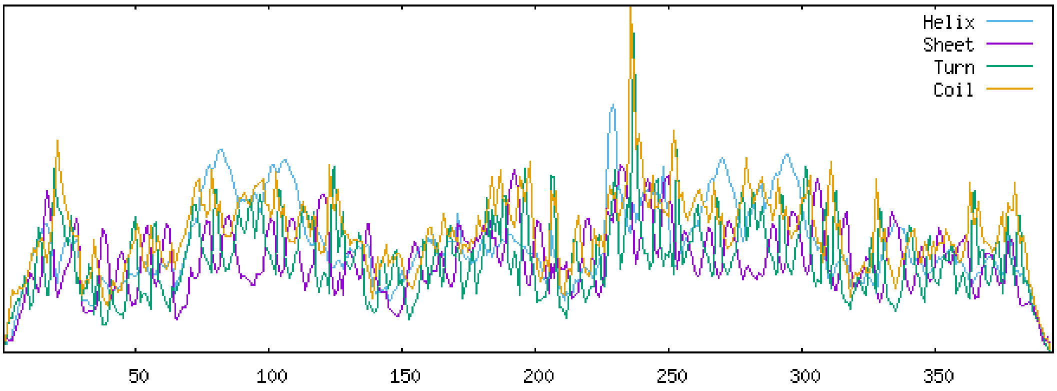

3. Nucleotide sequence of the SAM

SPusp45-Ps:

ATGAAAAAAAAGATTATCTCAGCTATTTTAATGTCTACAGTGATACTTTCTG
CTGCAGCCCCGTTGTCAGGTGTTTACGCTTTAGAAATTTCATCAACATGTGA
TGCT

CA:

ACTACTTATACCGTCAAATCTGGTGATACTCTTTGGGGAATCTCACAAAGAT
ATGGAATTAGTGTCGCTCAAATTCAAAGTGCGAATAATCTTAAAAGTACCAT
TATCTACATTGGTCAAAAACTTGTACTGACAGGTTCAGCTTCTTCTACAAAT
TCAGGTGGTTCAAACAATTCCGCAAGCACTACTCCAACCACTTCTGTGACA
CCTGCAAAACCAACTTCACAAACAACTGTTAAGGTTAAATCCGGAGATACC
CTTTGGGCGCTATCAGTAAAATATAAAACTAGTATTGCTCAATTGAAAAGTT
GGAATCATTTAAGTTCAGATACCATTTATATTGGTCAAAATCTTATTGTTTCA
CAATCTGCTGCTGCTTCAAATCCTTCGACAGGTTCAGGCTCAACTGCTACC
AATAACTCAAACTCGACTTCTTCTAACTCAAATGCCTCAATTCATAAGGTCG
TTAAAGGAGATACTCTCTGGGGACTTTCGCAAAAATCTGGCAGCCCAATTG
CTTCAATCAAGGCTTGGAATCATTTATCTAGCGATACTATTTTAATTGGTCAG
TATCTACGAATAAAA

Mtp：

TGTAAATCAACACATCCTTTATCATGTTCATTTCATCAATTACCTGCAAGAAG
TCCTTTACCATCATTAGATGCAGGACAATATGTTTTAGTTATGAAAGCAAATT
CAAGTTATTCAGGTAATTATCCATATTCAATTTTATTTCAAAAATTTTGA
